# The bHLH transcription factor VfTT8 underlies *zt2*, the locus determining zero tannin content in faba bean (*Vicia faba* L.)

**DOI:** 10.1038/s41598-020-71070-2

**Published:** 2020-08-31

**Authors:** Natalia Gutierrez, Carmen M. Avila, Ana M. Torres

**Affiliations:** Área de Genómica y Biotecnología, IFAPA-Centro Alameda del Obispo, Apdo 3092, 14080 Córdoba, Spain

**Keywords:** Genetics, Agricultural genetics, Gene expression, Genetic linkage study, Genetic markers, Genome, Genomics, Genotype, Haplotypes, Plant breeding, Plant genetics, Sequencing

## Abstract

Faba bean (*Vicia faba* L.) is an important protein-rich fodder crop, which is widely cultivated in temperate areas. However, antinutritional compounds such as condensed tannins, limit the use of this protein source in monogastric feed formulations. Previous studies demonstrated that two recessive and complementary genes, *zt1* and *zt2*, control absence of tannin and white flower colour in faba bean. An ortholog of the *Medicago* WD40 transcription factor *TTG1* was reported to encode the zt1 phenotype, but the responsible gene for zt2 is still unknown. Here we used a candidate gene approach combined with linkage mapping, comparative genomics and gene expression to fine map the *zt2* genomic region and to identify the regulatory gene controlling both traits. Seventy-two genes, including 23 MYB and bHLH regulatory genes predicted to be associated with anthocyanin expression together with WRKY proteins, were screened and genotyped in three mapping populations. The linkage groups constructed identified the regulatory gene, *TRANSPARENT TESTA8* (*TT8*), encoding a basic helix-loop-helix (bHLH) transcription factor, as the candidate for *zt2*. This finding was supported by qPCR analysis and further validated in different genetic backgrounds. Accordingly, *VfTT8* was downregulated in white flowered types while showing high levels of expression in wild genotypes. Our results provide new insights on the regulatory mechanisms of tannin biosynthesis in faba bean and will facilitate the development of an ultimate zt2 diagnostic marker for the fast generation of new value-added cultivars free of tannins and with improved nutritional value.

## Introduction

Since the beginnings of agriculture, legume species with high protein levels in seeds and foliage have been essential sources of dietary protein for humans and animals. Faba bean (*Vicia faba* L.) is a worldwide cultivated grain legume, known for its great yield potential and high protein content of 25–40%^1^. Nevertheless, the successful deployment of this protein rich fodder crop in feedstuffs for monogastric animals is severely limited by the presence of tannins, a class of polyphenols primarily located in the seed coat^2,3^, which are present at different concentrations depending on the variety, maturity, location and growth conditions^4^. Although tannins play an important role in plant growth and reproduction, providing protection against biotic and abiotic stresses^5^, they have a negative impact on nutritive value and digestibility, being responsible for decreases in feed intake, growth rate, feed efficiency, net metabolizable energy and protein digestibility in experimental animals^6^. Therefore, low-tannin content generally results in higher protein and energy digestibility for monogastric animals^7^. Consequently, the development of faba bean tannin-free cultivars is a key breeding objective to enhance the nutritional quality and broaden the use of this legume in the livestock feed industry.

Flavonoids are the most common group of polyphenolic compounds in the plant kingdom^8^. Thanks to the easily detectable mutant phenotypes in flower and seed pigmentation, flavonoid biosynthesis is one of the best-studied secondary metabolic pathways. These compounds play important roles in the interactions of plants with the environment, serving as pigments, signalling molecules, protectants against biotic and abiotic stresses as well as UV protection^9,10^. Proanthocyanidins (condensed tannins) and anthocyanins are major flavonoid end-products of a well conserved family of aromatic molecules, and both compounds are produced by related branches of the flavonoid pathway which utilize the same metabolic intermediates. Anthocyanin and tannin biosynthesis requires both the structural genes encoding the enzymes that participate in the formation of different flavonoids, as well as the regulatory genes that control transcription of the structural genes^11^. Thus, mutations in either structural or regulatory genes lead to loss of pigmentation in several plant species^12–15^.

Both structural and regulatory anthocyanin and tannin biosynthesis genes have been identified and cloned in model plants such as Arabidopsis, maize (*Zea mays*), snapdragon (*Antirrhinum majus*) and Petunia^9,16–18^. Highly conserved structural genes in this pathway include chalcone synthase (CHS), chalcone isomerase (CHI), flavanone 3-hydroxylase (F3H) and dihydroflavonol 4-reductase (DFR). Among the regulatory genes, DNA-binding R2R3-MYB transcription factors, basic-Helix-Loop-Helix (bHLH) transcription factors, and WD40 repeat proteins are known to form MYB-bHLH-WD repeat (MBW) complexes, which activate transcription of the structural genes in the anthocyanin pathway^19^.

In previous studies, we tested whether the faba bean genes responsible for the white flower and low tannin phenotypes function in the anthocyanin biosynthetic pathway. BLAST and multiple sequence alignment tools were used to design primers for amplification of the conserved key structural genes *CHS, CHI, F3H* and *DFR,* with the aim of identifying polymorphisms that might account for functional gene changes in contrasting faba bean populations. However, neither linkage analysis nor expression studies by RT-PCR pointed towards any of these genes as possible regulators of the zt-1 and zt-2 phenotypes^20^.

Studies in pea revealed, that the absence of flower pigmentation results from mutations in loci *A* and *A2,* encoding a bHLH transcription factor and a WD40 protein, respectively^21^. In lentils, the zero-tannin trait is controlled by a single recessive gene (*tan*) that encodes a bHLH transcription factor homologous to the *A* gene from pea^22^. In faba bean, zero-tannin (*zt*) is a simple character, which is governed independently by two complementary recessive genes, *zt1* and *zt2*^2,23^. These genes lead to a defect in synthesis of anthocyanins or their precursors at different steps in the pathway, resulting in plants with white flowers and zero tannin content^3,24^. Given the partial outcrossing behaviour of faba bean, crosses between zt1 and zt2 individuals will produce coloured plants with tannins that might contaminate the crop. For this reason, it is important to know which of the two genes is present in each tannin-free accession, in order to select the appropriate genitors in breeding programs.

Recently, a gene ortholog of the *Medicago* WD40 transcription factor *Transparent Testa Glabra 1* (*TTG1*), located on faba bean chromosome (chr.) II, was shown to encode the zt1 phenotype^25–28^. However, although several studies assigned the locus *zt2* to the distal part of chr. III^26,28,29^, the responsible gene is still unknown. More recently, the latter authors^29^ reported that a KASP (Kompetitive Allele‐Specific PCR) marker (SNP marker Vf_Mt7g100500_001), located at 10.5 cM from the *zt2* locus, could be used as a reliable marker to discriminate low‐tannin faba bean plants carrying *zt2*. However, the relatively high genetic distance from the *zt2* locus bears the risk of false selection, due to possible recombination events. Therefore, marker enrichment of the genomic region is needed to identify the underlying gene and allow a fully reliable marker-assisted selection in breeding programs.

The goal of the present study was to saturate the *zt2* genomic region and to identify the candidate gene for the white flower trait, using a combination of genetic linkage, association studies and comparative genomics with the model legume species *Medicago truncatula* (https://www.medicagohapmap.org/fgb2/gbrowse/mt35/). Upon confirmation of the chromosomal region, we exploited the extensive collinearity between faba bean and *Medicago* to saturate the target region and mine candidate genes potentially associated with the trait. This approach pinpointed a number of MYB and bHLH regulatory genes potentially associated with the anthocyanin expression. Once the gene sequences were identified, we searched for mutations associated with the target phenotypes, using different mapping populations and genetic backgrounds.

In summary, here we describe a detailed approach for fine mapping, identification and validation of the gene underlying *zt2* in faba bean. This result will allow the development of a highly efficient diagnostic marker for the selection of zt2 cultivars free of anti-nutritional compounds.

## Material and methods

### Plant material and sample collection

Three mapping populations (two F_2_ and one F_7_) segregating for tannin content (TC) and flower colour (FC), wild spotted type vs. white flower, were used in this study: (1) M × D: 50 F_2_ individuals originated from the cross of MAYA (M), with spotted flowers and high TC and DISCO (D), a white flower/zero tannin inbred line, carrying the *zt2* gene, (2) W × D: 56 F_2_ individuals derived from the cross of WIZARD (W), a wild-type flower line with high TC and DISCO (D) and, (3) Vf6 × zt2: 62 F_7_ individuals, where the maternal parent (Vf6) has spotted flowers and high TC while zt2 is a tannin-free white flowered line. The populations were selfed at the IFAPA of Córdoba, Spain. Plants were grown in insect proof cages to avoid outcrossing. Young leaves were collected from all samples and DNA was isolated according^32^. The concentration and purity of DNA was measured by a NanoDrop ND-1000 spectrophotometer. The final concentrations of all DNA samples were adjusted to 10 ng/µl for high-throughput genotyping.

### Phenotypic evaluation

F_2_ plants from crosses M × D and W × D were selfed to the F_3_ generation and the FC was recorded at each F_3_ family (15 plants per line) to infer their corresponding genotype (homozygous or heterozygous). In the RIL population (Vf6 × zt2), FC was scored in all the lines self-pollinated from F_2_ to F_7_. Segregation ratios were tested against the expected ratio using a chi-square analysis for goodness of fit. Phenotypic data on FC in the three populations are available in the Table 1.

**Table 1:** Segregation of flower colour (FC) in the three populations used in this study.

Population	Generation	Individuals evaluated	Observed ratio	Expected ratio	*χ*^2^	*p* value*
M × D	F_2:3_	50	12:30:8	1:2:1	2.64	0.27
W × D	F_2:3_	56	18:24:14	1:2:1	1.71	0.42
Vf6 × zt2	F_7_	62	36:26	1:1	1.61	0.20

Observed and expected ratios correspond to the homozygous wild type: heterozygous: homozygous white flower colour in F_2:3_ generations and homozygous wild type: homozygous white flower colour in F_7_ RIL population.

*α = 0.5.

### Genomic location and saturation methodology

Previous linkage map approaches ascribed the target *zt2* region in the distal part of faba bean chr. III between the ESTs markers Pis-Gen-8-1-1 and LG38^26,28,30,31^. Using the sequence information deposited in the *M. truncatula* Genome Database (MTGD) (https://www.medicagogenome.org/), these genes corresponded to Medtr1g040675 and Medtr1g087900, respectively, spanning 24.3 Mb.

For marker saturation and candidate gene identification we performed a detailed genomic analysis of this syntenic region. Seventy-two genes, including regulatory genes (MYB and bHLH) predicted to be associated with anthocyanin expression together with WRKY proteins were selected to be screened for polymorphism in the three mapping populations (Supplementary Table S1).

### Sequencing and genotyping technique

The 72 genes selected include six ESTs markers previously assigned to the target interval^26,31^ and 23 newly designed *M. truncatula* markers associated with the anthocyanin expression. The remaining 42 genes were genotyped using the Kompetitive Allele-Specific PCR (KASPar) assays developed by Webb^27^ and provided by LGC genomics (https://www.biosearchtech.com/products/pcr-kits-and-reagents/genotyping-assays/kasp-genotyping-chemistry) (Supplementary Table S1).

Different tools, including BLASTn and multiple sequence alignment analysis were used to design primers suitable for DNA amplification. The sequences were BLASTed against an in-house faba bean transcriptome^33^ to obtain the orthologous sequence in faba bean. For intron–exon boundary prediction and primer design, alignment between sequences were implemented in Geneious v.7.1.5 (https://www.geneious.com) The information about primer sequence, annealing temperature, marker type, orthologous sequence in faba bean, locus name and annotation in *M. truncatula* of the 23 new markers are available in Supplementary Table S2.

To obtain the sequences from DNA samples (parental lines and two contrasting individuals from each population), PCR reactions were conducted in a total volume of 25 µl, using 5 µl DNA of each sample, 200 nM of each primer, 2 mM MgCl2, 200 µM dNTP and 0.6 U Taq polymerase Biotools (B&M Labs, S.A., Madrid, Spain). Amplification products were purified using a standard protocol for DNA precipitation with sodium acetate and ethanol (1/10 3 M sodium acetate, 2 v/v ethanol) (https://www.thermofisher.com/es/es/home/references/protocols/nucleic-acid-purification-and-analysis/dna-protocol/sodium-acetate-precipitation-of-small-nucleic-acids.html#comergent_product_list_15878). PCR products were sequenced by Sanger at STABVIDA (Caparica, Portugal). For each sample, four PCRs were done, and the repeated products were then mixed for sense and antisense strand sequencing.

Sequence analysis and identification of polymorphisms were conducted using Geneious v.7.1.5. For each gene, the sense and antisense sequences were aligned and the consensus sequence further analyzed with BLASTn against the *M. truncatula* genome (Mt4.0) to assign the percent-consensus-identity. Sequence polymorphisms between the samples were transformed to CAPS (Cleaved Amplified Polymorphism) markers by restriction enzyme digestion of the PCR products. The digestions were carried out in a 15 µl volume of PCR using 5 µl of the amplified products and 3U of the respective restriction endonuclease (ThermoFisher Scientific). The digestions were incubated overnight at their respective temperature. The restricted fragments were separated in 2% agarose gel. Detailed information of the specific gene-based markers designed in this study together with the KASPar markers assayed, is shown in the Supplementary Table S1.

### Linkage maps calculation

The segregation of each marker was tested for goodness of fit to the expected segregation ratio (1:2:1 in the two F_2_s families and 1:1 ratio in the RIL population), using the chi-square test. Linkage maps were constructed using JoinMap 4.1^34^. Markers were grouped using regression mapping algorithm option at a minimum LOD score threshold of 4 and maximum recombination frequency of 0.4 as general linkage criteria to establish linkage groups (LG). Recombination fractions were converted to centimorgans (cM) using the mapping function of Kosambi^35^.

### qPCR samples, RNA isolation, cDNA synthesis and primer design

The most important guidelines of the MIQE checklist^36^ were considered according to the practical approach for quantitative real-time PCR (qPCR) experiments proposed by Taylor^37^. The expression profile of candidate genes was analysed in pigmented and white flowers from the Vf6 × zt2 and M × D populations at two different developmental stages. Stage 1 (S1) included immature flowers buds of approximately 1.5 cm length while the stage 2 (S2) included young flowers of 2 cm length and black colour apparent at the top of the petal in the wild types. CYP2 and ELF1A, previously reported as the most stable genes for normalization of the gene expression in the TC experiment were used as reference^38^.

Only petal tissue was used for total RNA extraction. In order to minimise variation in gene expression among individual plants and stages, petal tissue from three individuals were collected and pooled together for RNA isolation. Finally, three pools of biological replicates were used per individual genotypes. Each sample was frozen in liquid nitrogen and stored at – 80 °C until RNA extraction. In short, the experimental design consisted in a total of 24 samples (2 genotype × 2 stages of development × 2 technical repetitions × 3 biological repetitions).

The Direct-zol RNA Kit (Zymo Research) was used to isolate a high-quality RNA directly from samples in TRIzol Reagent. RNA concentration was determined by measuring the optical density using a NanoDrop spectrophotometer. Only the RNA samples with A_260_/A_280_ ratio between 1.9 and 2.1 and A_260_/A_230_ greater than 2.0 were used in the analysis. cDNA of each sample was obtained by iScript cDNA Synthesis kit (Bio-Rad, Hercules, California) and diluted to a concentration of 20 ng/µl. A pooled sample comprising all samples considered in the experiment was included for each gene as inter-run calibrator to detect and correct inter-run variation. No-template controls were also included.

Specific primer pairs were designed using the following criteria: Tm of 60 ± 1 °C and PCR amplicon lengths of 80–120 bp, yielding primer sequences with lengths of 19–26 nucleotides and GC contents of 40–80%. Designed primers were synthesized by Integrated DNA Technologies (Leuven, Belgium). Detailed information on the primers designed for RT-qPCR is shown in Table 2.

**Table 2:** Information on RT-qPCR primers used in this study.

GenBank accession	Medicago locus	Annotation	Primer sequence (5′–3′)	Gene position	PCR product size (bp)	PCR efficiency	PCR product Tm (°C)
KP006493.1	MTR_1g022445	*Vicia faba* dihydroflavonol reductase 1 (DFR1)	TCTTGTTTGAGCATATGGAAGTTGAG	Exon 5	111	1.94 ± 0.06	74.12 ± 0.06
			GGACATTGTACTCTGGAAATTTTGCA				
XM_003590588.3	MTR_1g072140	Transducin/WD40 repeatprotein	TCCGACTCCCACTACGTCTGC	Exon 1	108	1.93 ± 0.03	84.69 ± 1.01
			GATGTGTGTATGGCCACGGAG				
XM_024786476.1	MTR_1g072320	Transcription factor bHLH (TT8)	CGGAGAGACAAATCCGCCAAC	Exon 2	117	1.94 ± 0.02	78.99 ± 0.35
			CCGACACCAGGAGGAAATGAG				
XM_003590628.3	MTR_1g072530	Transcription factor bHLH66	CAAGCTTACATACTGGACGACATC	Exon 5–6	110	1.91 ± 0.03	75.90 ± 0.27
			ACTAGTGGTATAGCATTTGAGTCA				
XM_013613397.2	MTR_1g077640	bHLH transcription factor	TAGGAAGTGTGATCAAATATGCAGAC	Exon 7–8	120	1.94 ± 0.03	77.56 ± 0.31
			CTTCAAATGCCCATGTAGCACC				

GenBank accession, medicago locus, annotation NCBI, primer sequences, gene position and amplicon size. Primer PCR efficiency and PCR product Tm data represent mean values ± sd.

### Real time qPCR assays

The qPCR was carried out using the iTaq Universal SYBR Green Supermix on an ABI PRISM 7500 Real Time PCR System (Applied Biosystems, Foster City, CA, USA). Experiments were performed in 96-well optical reaction plates (P/N 4306737) with MicroAmp optical adhesive film (P/N 4311971) from Applied Biosystems. A master mix with total volume of 11 µl for each PCR run was prepared, containing 5 µl of diluted cDNA (10 ng/µl), 5 µl of iTaq Universal SYBR Green Supermix (Bio-Rad, Hercules, California) and a primer pair with a concentration of 0.22 µM each. The PCR conditions were 95 °C for 10 min followed by 40 cycles at 95 °C for 15 s and 60 °C for 1 min. The specificity of the of the PCR products was confirmed by single and sharp peaks at the melting curves analysis after 40 amplification cycles.

Fluorescence was analyzed using 7500 Software v2.0.1. All amplification plots were analyzed using a threshold values of 0.2 to obtain Cq (quantification cycle) values for each gene-cDNA combination. PCR efficiency of each primer pair was determined for all samples by LinRegPCR program v.11^39^, using raw normalized (Rn) fluorescence as input data.

The relative gene expression (RGE) was calculated using the advanced quantification model with efficiency correction, multiple reference genes normalization and use of error propagation rules described by Hellemans^40^ (Eq. 1), where RQ = E^∆Ct^, being E = PCR efficiency for each primer used to amplified each target gene (TG) and Ct = the number of cycles needed to reach 0.2 arbitrary units of fluorescence. The two reference genes (RG) used for data normalization were CYP2 and ELF1A^38^.

1 $$RGE=\frac{RQ{\text{TG}}}{Geomean\lceil RQRG\rceil }.$$

The RGE values were log-transformed and the significance values to determine differences of the expression of genes were obtained by the ANOVA test using the R programming language.

## Results

### Segregation analysis of flower colour (FC)

Of the 50 F_2:3_ plants screened from the M × D population, 12 had spotted flower, 30 were heterozygous/spotted and 8 showed white flower, fitting a 1:2:1 ratio. Further, the 56 F_2:3_ families from cross W × D, also exhibited a good fit for the expected 1:2:1 (spotted: segregating: white) ratio. Similarly, the 62 RILs evaluated fit the expected 1:1 (spotted: white) ratio (Table 1). The results from FC segregation in these populations further confirmed the monogenic control of the gene with a dominant *ZT2* (spotted flower) and a recessive *zt2* (white flower) allele.

### Genotyping and linkage analysis

The collinearity between faba bean and the model *Medicago* was exploited to develop new markers and to fine map *zt2*, in the distal region of faba bean chr. III. Seventy-two gene markers, including candidate genes encoding MYB, bHLH and WRKY proteins, were surveyed in the interval flanked by markers Medtr1g040675 and Medtr1g087900 (Supplementary Table S1). Six of these were ESTs previously used by Satovic et al.^31^. Moreover, we designed 23 new primer pairs in genes putatively associated with anthocyanin expression, while the remaining 43 genes were genotyped using the KASPar technique.

Thirty out of the 72 markers tested (23 KASP-SNPs, 6 CAPs, 1 Amplified Length Polymorphism/ALP) revealed polymorphisms in at least one of the three faba bean populations, and 12 of these were anchor markers common among populations (Supplementary Table S1). Five of the 23 candidate genes potentially associated with anthocyanin expression exhibited polymorphisms and were thus further genotyped and mapped. The most polymorphic population was M × D with 20 markers, followed by Vf6 × zt2 with 15 markers and by W × D with 13 markers.

**Figure 1:**
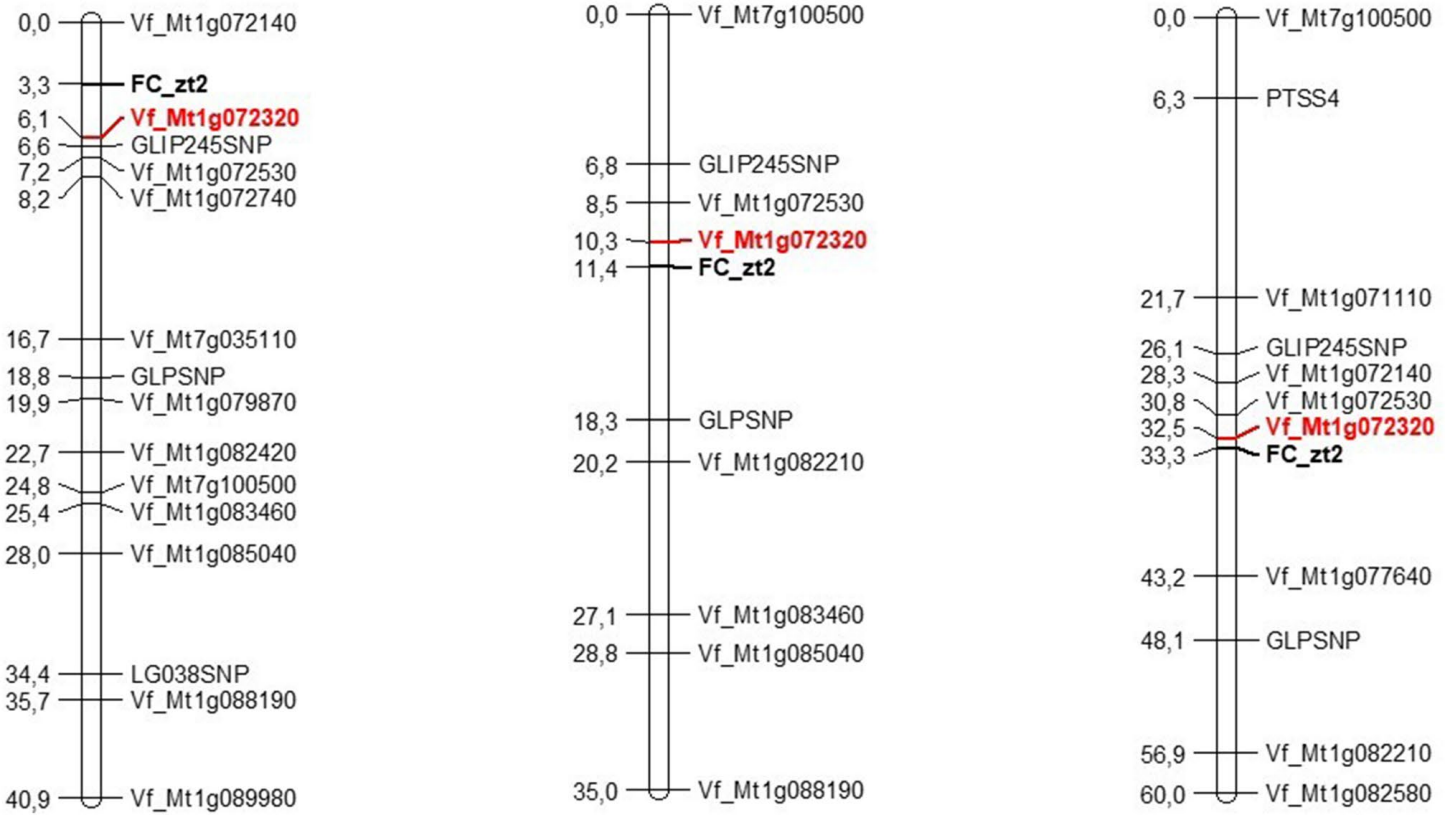
Linkage maps from three faba bean populations segregating for flower colour and carrying the gene *zt2*. M × D F_2_ population (left), W × D F_2_ population (middle) and Vf6 × zt2 RIL population (right). Marker names are on the right and the estimated map distances are on the left. Recombinant fractions were converted to centiMorgans using the mapping function of Kosambi.

Three linkage groups were constructed (Fig. 1). Fine mapping analysis showed that in cross M × D, *zt2* is localized in a window of 6.1 cM between markers Vf_Mt1g072140 and Vf_Mt1g072320, whereas in W × D, *zt2* is in a window of 8 cM between Vf_Mt1g072320 and GLPSNP (MTR_1g079490). Finally, in the RIL population (Vf6 × zt2), *zt2* was flanked by Vf_Mt1g072320 and Vf_Mt1g077640 (Fig. 1). We found high collinearity between faba bean chr. III and *M. truncatula* chr. I, except for two markers Vf_Mt7g035110 and Vf_Mt7g100500 which revealed some chromosomal rearrangements due to insertions from Mt Chr. 7. All markers maintained a similar mapping order in the three populations. The closest marker to FC in the three populations (2.8 cM, 1.1 cM and 0.8 cM, respectively) was Vf_Mt1g072320, corresponding to the bHLH transcription factor *TRANSPARENT TESTA8*^42^ henceforth referred to as VfTT8. We thus selected VfTT8 as the best candidate for *zt2* in *V. faba*.

### Haplotype analysis

During fine mapping, 168 individuals (50 F_2:3_ from M × D, 56 F_2:3_ from W × D and 62 RILs from cross Vf6 × zt2) were screened to identify recombinants among flanking markers (Supplementary Table S3). Two individuals in W × D and Vf6 × zt2, or three in M × D, showed at least one recombination event between *zt2* and the closest marker Vf_Mt1g072320. A number of 5–9 crossovers were also observed in the rest of flanking markers and crosses, except for Vf_Mt7g100500, which was previously reported by Zanotto^29^ as a reliable marker for the selection of *zt2.* Strikingly, marker Vf_Mt7g100500 revealed the highest number of misclassified individuals in the three populations (17, 10 and 20, respectively). This finding contradicts the results of the previous report^29^ and precludes the reliable use of this marker in faba bean breeding programs. We concluded that, according to these recombination events, Vf_Mt1g072320 was the best candidate to discriminate low tannin individuals carrying the *zt2* gene.

In the case of Vf_Mt1g072320, we designed two primer pairs in different genomic regions in order to ensure amplification in different genetic backgrounds and to optimize resolution of the banding patterns (Supplementary Table S2). Using primer pair 1, the recessive allele linked with the *zt2* locus in crosses W × D and Vf6 × zt2 is *G.* Therefore, considering identity by descent in the germplasm analysed, and without recombination between the marker and the *zt2* locus, white flower genotypes are expected to be *GG* whereas wild flower genotypes are expected to be *AG* or *AA.* Primer pair 2 was only used in cross M × D, and the corresponding genotypes were CC in white flower zt2 genotypes and *TC* or *TT* in wild individuals (Supplementary Table S3).

### Expression analysis of candidate genes

To corroborate that VfTT8 corresponds to the *zt2* gene, RT-qPCR analysis was performed on flower tissue of two RIL individuals derived form from cross Vf6 × zt2 with contrasting FC. The candidate genes assayed were those most closely linked to FC_*zt2* in the three linkage maps (see Fig. 1): the DFR1 gene MTR_1g022445 (reported by Ray^41^), the WD40 protein MTR_1g072140, and the bHLH transcription factors MTR_1g07230 (*VfTT8*), MTR_1g072530 and MTR_1g077640. The cDNAs from RIL6 (pigmented flower) and RIL2 (white flower) obtained from two different developmental stages (S1 and S2) were analysed.

To confirm the sensitivity and specificity of RT-qPCR, we tested the presence of genomic DNA (gDNA) contamination in the cDNA samples using primer pairs for MTR_1g072530 and MTR_1g077640, which span an exon-exon junction (Table 2). The first primer pair amplified a 195 bp product using gDNA as template or a 110 bp band using cDNA, while the second primer amplified a 224 bp or a 120 bp band using gDNA or cDNA, respectively. None of the cDNAs tested produced bands corresponding to residual gDNA, confirming the purity of samples. The dissociation curve analysis confirmed that all the primer pairs used produced a single and specific PCR product (Table 2). Mean PCR efficiency for all genes was up to 95.5%, while that for the reference genes *CYP2* and *ELF1A* was 1.88 ± 0.009 and 1.88 ± 0.01 (mean ± sd), respectively.

**Figure 2:**
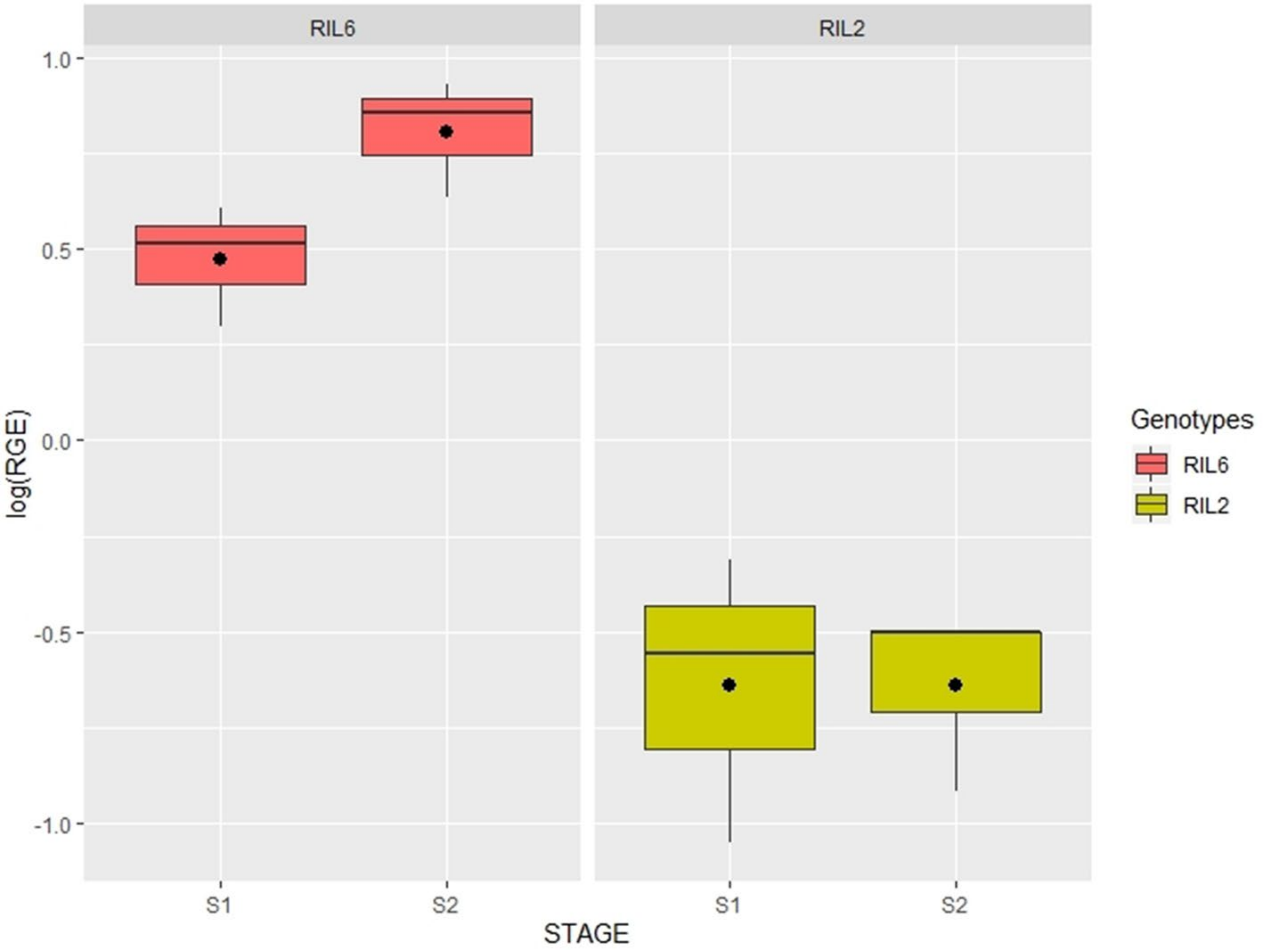
Boxplots showing transcript levels of MTR_1g072320 in RIL6 (pigmented flower) and RIL2 (white flower) from cross V6 × zt2, in two developmental stages: immature flowers (S1) and young flowers (S2). Relative gene expression was log-transformed as detailed in “Material and methods” section. Expression values are shown as median (horizontal line), mean (asterisk), upper and lower quartiles (box) and range (whiskers).

**Figure 3:**
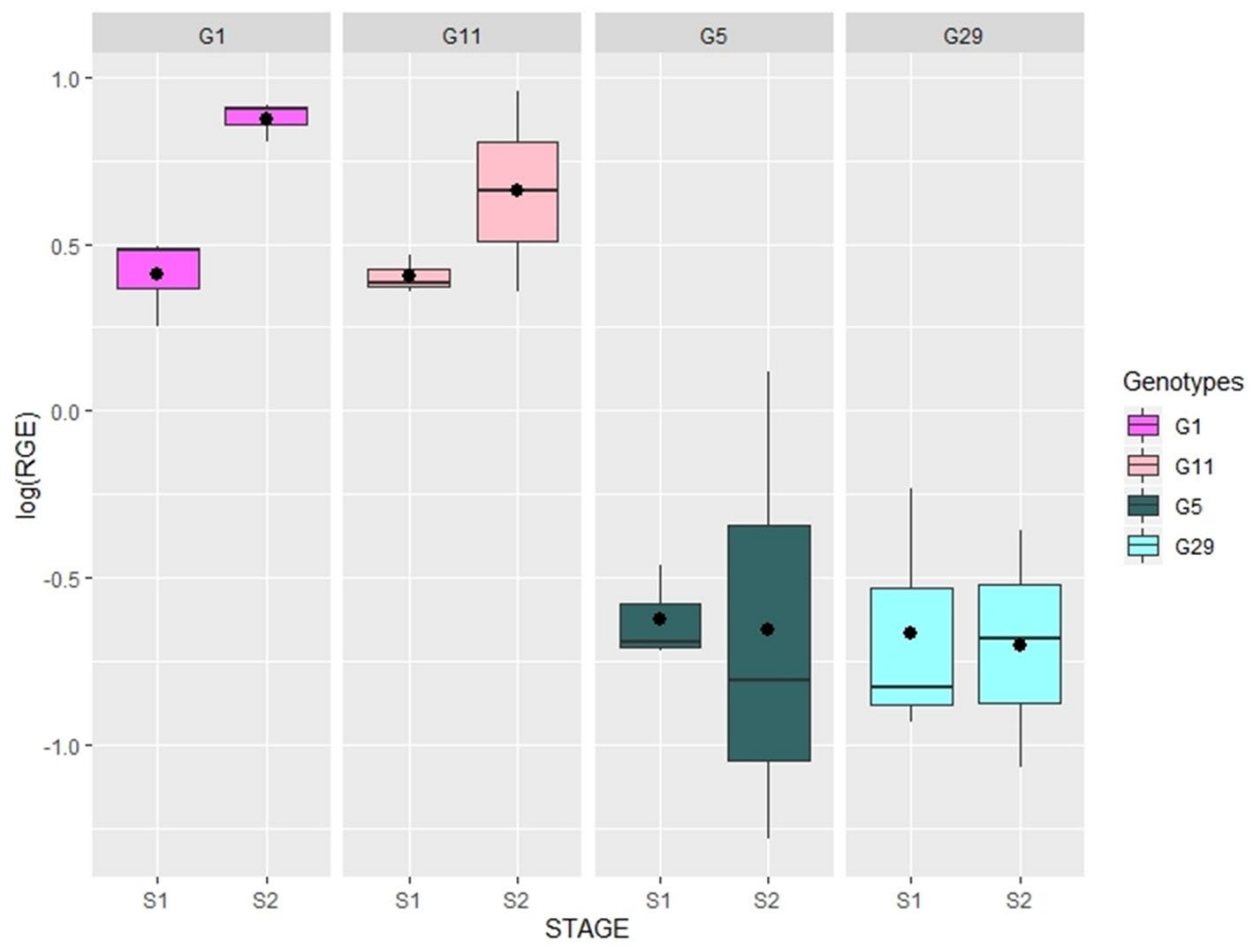
Validation of *MTR_1g072320* expression values in the F2 population M × D. Boxplots show transcript levels in two individuals with pigmented flower (G1, G11) and two white flowers (G5, G29), in two developmental stages: immature flowers (S1) and young flowers (S2). Relative gene expression was log-transformed as detailed in “Material and methods” section. Expression values are shown as median (horizontal line), mean (asterisk), upper and lower quartiles (box) and range (whiskers).

The overall expression pattern of MTR_1g072320 in both flowering stages was significantly higher (*p* < 0.001) in the V6 × zt2 wild-type line (RIL6) compared to RIL2, being strongly down-regulated in this white flowered genotype (Table 3 and Fig. 2). To validate these results, the expression of MTR_1g072320 was measured in a second faba bean population (M × D), using cDNA from two F2 individuals (G1 and G11) with pigmented and two (G5 and G29) with white flowers. The expression pattern of MTR_1g072320 matched that obtained in the Vf6 × zt2 RILs, showing highly significant differences in the expression level (*p* < 0.001) between the contrasting FC genotypes. Collectively, these results revealed a strong down-regulation of MTR_1g072320 in white flowered genotypes, further confirming that *VfTT8* is the gene responsible for loss of flower pigmentation and absence of tannins in the *zt2* genotypes (Table 4; Fig. 3).

**Table 3:** Gene expression ratios in RIL population Vf6 × zt2.

Medicago locus	RIL population Vf6 × zt2			
	RIL6 (wild type)		RIL2 (zt2)	
	Stage 1	Stage 2	Stage 1	Stage 2
MTR_1g022445	0.08	0.28	− 0.18	− 0.19
MTR_1g072140	− 0.49	− 0.09	− 0.42	− 0.16
MTR_1g072320	**0**.**47**	**0**.**81**	− **0**.**64**	− **0**.**64**
MTR_1g072530	− 0.03	0.08	− 0.02	− 0.04
MTR_1g077640	0.35	− 0.50	0.06	0.09

Values indicate log average expression ratios of three biological replicates from each of the genotypes used in the study. RIL6 (wild type) and RIL2 (mutant type) are F_7_ individuals from the Vf6 × zt2 population. Statistically significant differences in expression (*p* < 0.001) are indicated in bold.

**Table 4:** Expression of the candidate gene *MTR_1g072320* in four F2 genotypes from the M × D population.

Medicago locus	F2 population Maya × Disco (MxD)							
	G1 (wild type)		G11 (wild type)		G5 (zt2)		G29 (zt2)	
	Stage 1	Stage 2	Stage 1	Stage 2	Stage 1	Stage 2	Stage 1	Stage 2
MTR_1g072320	0.41	0.88	0.40	0.66	− 0.63	− 0.66	− 0.67	− 0.71

Values indicate log average expression ratios of three biological replicates from each of the genotypes G1 and G11 (wild type); and G5 and G29 (mutant). All the samples showed statistically significant differences in regulation (*p* < 0.001).

## Discussion

Faba bean is a valuable protein crop with a high potential for the feed industry to shift towards more sustainable raw materials. However, despite its prominent nutritional value, faba beans contain antinutrients such as condensed tannins, which are present in the seed coats of coloured-flowering faba bean varieties, reducing the bioavailability of proteins and minerals. The zero-tannin (*zt*) trait is governed independently by the two complementary recessive genes *zt1* and *zt2.* Gene *TTG1* encodes the zt1 phenotypes^25–28^, but the gene underlying zt2 was still unknown. Thus, identifying markers closely linked to *zt2* has been a key objective to increase the accuracy and efficiency of selection and facilitate marker-assisted breeding of faba bean tannin-free cultivars. Previous studies had adscribed this locus to the distal part of chr. III^26,28,29^, and the latter authors also reported KASP SNP marker Vf_Mt7g100500_001, located at 10.5 cM from flower colour, as being successfully used to discriminate low tannin plants, even though the closest flanking marker Vf_Mt1g072740_001, which is only at 3 cM distance, failed to distinguish these genotypes in the validation panel^29^. In contrast to the previous report, we found here that Vf_Mt7g100500_001 produced the highest number of misclassified individuals for any of the faba bean populations assayed, thus preventing its use in marker assisted breeding.

We set out to identify candidate transcription factors responsible for the unpigmented *zt2* genotypes using marker enrichment of the target region. For the first time, a set of 23 MYB, bHLH and WD40 genes present in the collinear *M. truncatula* region were assayed. Because not all markers are applicable across populations due to lack of polymorphism, the use of multiple mapping populations of diverse genetic backgrounds is important for accurate fine-mapping and, ultimately, target gene identification. To maximize genetic diversity, we analysed three faba bean segregating populations, thereby increasing the level of polymorphisms and narrowing down the search of potential candidates to a much shorter segment of the *M. truncatula* genome. This analysis identified Vf_Mt1g072320, encoding a TT8 transcription factor, as the candidate gene controlling zero tannin zt2 in *V. faba*.

In *Arabidopsis*, *TT8* encoding a bHLH domain transcription factor, *TTG1* encoding a WDR protein, and *TT2* encoding a R2R3 MYB domain protein, have been identified as key determinants for proanthocyanidin accumulation in developing seeds^42,43^. The MYB–bHLH–WD40 (MBW) complex TT2–TT8–TTG1 activates the transcription of structural genes in the anthocyanin pathway and thus controls the accumulation of condensed tannins in the *Arabidopsis* seed coat^19^. In Medicago, both WD40 repeat proteins and bHLH transcription factors (MtTT8) are involved in the regulation of anthocyanin and proanthocyanidin biosynthesis^42–45^. In pea, the absence of pigmentation in the flower is the result of mutations in loci *A* and *A2,* encoding a bHLH transcription factor and a WD40 protein, respectively^21^. A single recessive gene, *tan*, encoding a bHLH transcription factor homologous to that encoded by the *A* gene in pea^22^, controls white flower in lentils, while in faba bean the WD40 transcription factor *VfTTG1* is responsible for the zt1 phenotypes^26,28,29^.

The broad conservation of the MBW complex controlling anthocyanin pigmentation in Eudicots^44,46^ suggests that other MYB or bHLH transcription factor should control the *zt2* gene in faba bean. In the present study, *MtTT8* (Mt1g072320), a Medicago ortholog of the *A* and *tan* genes of pea and lentil, respectively, was assayed as a promising candidate for determining low TC in faba bean. *VfTT8* (Vf_Mt1g072320) was polymorphic in the three faba bean populations tested, and strongly cosegregated with white flower lines, supporting the identity of the candidate. These results were further confirmed by RT-qPCR analyses in different populations and genetic backgrounds, revealing that *VfTT8* expression levels were very low in white flowered types, in contrast to high transcript levels detected in the wild genotypes.

From the data presented here, we conclude that *VfTT8* is the gene responsible for the zt2 phenotypes in faba bean. Although further analysis is necessary to elucidate the mechanistic basis for the observed down-regulation of the gene in white flowered types, our results open the door to the development of an ultimate diagnostic marker based on the allelic variant causing this phenotypic effect. This study therefore increases our understanding of the regulatory mechanisms underlying tannin biosynthesis in this crop and will allow the development of a fast and reliable tool for the generation of value-added faba bean cultivars with optimized flavonoid content.

## Supplementary information

Supplementary Tables.

## Data Availability

The datasets generated and analyzed during the current study are available from the corresponding author on request.
