# Supplementary material for: The bHLH transcription factor VfTT8 underlies zt2, the locus determining zero tannin content in faba bean (Vicia faba L.)
Source: Sci Rep. 2020 Aug 31;10:14299. doi: 10.1038/s41598-020-71070-2 (PMC7459296; doi:10.1038/s41598-020-71070-2)
Supplement: Supplementary file 1 — Supplementary Tables. [file 41598_2020_71070_MOESM1_ESM.pdf]

The bHLH transcription factor VfTT8 underlies *zt2, the locus determining zero tannin content in faba bean (Vicia faba L.)*

Natalia Gutierrez[1*], CM Avila[1], Ana M. Torres[1]
[1] Área de Genómica y Biotecnología, IFAPA-Centro Alameda del Obispo, Apdo 3092, E-14080 Córdoba, Spain.
*Corresponding author e-mail: natalia.gutierrez.leiva@juntadeandalucia.es

Supplementary Table S1. Molecular markers surveyed in faba bean, derived from the syntenic zt2 region (Medtr1g040675-Medtr1g087900). The table shows the name given to each marker, the corresponding Medicago locus in NCBI, the gene description, the orthologous locus in faba bean (after BLASTing against the in-house faba bean transcriptome), the genotyping technique used and the result after sequence analysis and genotyping in three segregating populations.

Name	Medicago locus NCBI	Gene description	BLASTn_VF_Transcriptome (Ocaña et al. 2016)	Genotyping technique	Sequence analysis	Markers mapped in M x D*	Markers mapped in W x D*	Markers mapped in 6 x ZT2*
PISGEN_8_1_1[a]	MTR_1g040675	Auxin response factor	-	ER digestion	Monomorphic	-	-	-
**Vf_Mt1g043080**[b]	MTR_1g043080	MYB relation transcription (MYB)	Contig15498	ER digestion	ER not available	-	-	-
Vf_Mt1g044570[c]	MTR_1g044570	CAX-interacting protein	-	KASPAR assay	Polymorphic	-	-	X
**Vf_Mt1g045610**[b]	MTR_1g045610	R2R3-MYB Transcription factor	No hits found	ALP	Polymorphic	X	X	X
Vf_Mt1g045800[c]	MTR_1g045800	G3BP-like protein	-	KASPAR assay	Monomorphic	X	-	-
mtmt_GEN_01090_01_1	MTR_1g050520	Protein kinase and PP2C-like domain-containing protein	-	ER digestion	Monomorphic	-	-	-
Vf_Mt1g050730[c]	MTR_1g050730	Pre-mRNA-processing ATP-dependent RNA helicase prp5	-	KASPAR assay	Polymorphic	-	X	-
Vf_Mt1g056180[c]	MTR_1g056180	uncharacterized LOC11431185	-	KASPAR assay	Polymorphic	X	-	-
cgP137FSNP[c]	MTR_1g056180	uncharacterized LOC11431185	-	KASPAR assay	Polymorphic	-	-	X
Vf_Mt1g061800[c]	MTR_1g061800	Threonine dehydratase biosynthetic, chloroplastic	-	KASPAR assay	Monomorphic	-	-	-
Vf_Mt1g064060[c]	MTR_1g064060	Adenosine kinase 2	-	KASPAR assay	Monomorphic	-	-	-
PTSS4[a]	MTR_1g069105	26S proteasome regulatory subunit 4 homolog A	-	ER digestion	Polymorphic	-	-	X (MaeI)
**Vf_Mt1g070180**[b]	MTR_1g070180	bHLH transcription factor	Locus_34012_Transcript_1/1_Confidence_1.000_Length_809	ER digestion	Monomorphic	-	-	-
**Vf_Mt1g070455**[b]	MTR_1g070455	WRKY family transcription factor	Locus_48067_Transcript_1/1_Confidence_1.000_Length_887	ER digestion	Monomorphic	-	-	-
**Vf_Mt1g070870**[b]	MTR_1g070870	bHLH transcription factor	Contig4759	ER digestion	Monomorphic	-	-	-
Vf_Mt1g071110[c]	MTR_1g071110	2-methyl-6-phytyl-1,4-hydroquinone methyltransferase,	-	KASPAR assay	Polymorphic	-	-	X
GLIP245SNP[c]	MTR_1g071430	Protein LIFEGUARD 4	-	KASPAR assay	Polymorphic	X	X	X
PISGEN_25-2-3-1[a]	MTR_1g071610	Acetyl-CoA carboxylase, putative	-	ER digestion	Monomorphic	-	-	-
**Vf_Mt1g072130**[b]	MTR_1g072130	PHDfingerprotein,putative	Locus_31961	ER digestion	Monomorphic	-	-	-
**Vf_Mt1g072140**[b]	MTR_1g072140	Transducin/WD40 repeatprotein	No hits found	ER digestion	Polymorphic	X (SgrAI)	-	X(PfeI)
**Vf_Mt1g072320**[b]	MTR_1g072320	Transcription factor bHLH (TT8)	No hits found	ER digestion	Polymorphic	X (Eco130I/TasI)	X (DraI)	X (DraI/RsaI)
**Vf_Mt1g072530**[b]	MTR_1g072530	Transcription factor bHLH66	Locus_21144	ER digestion	Polymorphic	X (NdeI)	X (SmoI)	X (Tetra-arm)
Vf_Mt1g072640[c]	MTR_1g072640	Protein PALE CRESS, chloroplastic	-	KASPAR assay	Monomorphic	-	-	-
Vf_Mt1g072740[c]	MTR_1g072740	Pre-mRNA-processing factor 17	-	KASPAR assay	Polymorphic	X	-	-
Vf_Mt1g073000[c]	MTR_1g073000	Polyadenylate-binding protein 1	-	KASPAR assay	Monomorphic	-	-	-
**Vf_Mt1g073170**[b]	MTR_1g073170	MYB related transcription factor	Locus 29372	ER digestion	Monomorphic	-	-	-
**Vf_Mt1g073920**[b]	MTR_1g073920	Actin related rprotein ARP4	Contig21805	ER digestion	ER not available	-	-	-
**Vf_Mt1g074370**[b]	MTR_1g074370	Ethylene-responsive transcription factor	Contig5707	ER digestion	Unreliable data for genotyping	-	-	-
Vf_Mt1g075140[c]	MTR_1g075140	DNA-directed RNA polymerase II subunit RPB7	-	KASPAR assay	Monomorphic	-	-	-
**Vf_Mt1g075320**[b]	MTR_1g075320	N-carbamyl-L-amino acid amidohydrolase	Locus_2084_Transcript 2180	ER digestion	Monomorphic	-	-	-
Vf_Mt1g075610[c]	MTR_1g075610	Cell division control protein 2 homolog D	-	KASPAR assay	Monomorphic	-	-	-
**Vf_Mt1g075740**[b]	MTR_1g075740	COP1-interacting-like protein	Contig16561	ER digestion	ER not available	-	-	-
**Vf_Mt1g076150**[b]	MTR_1g076150	MYB related transcription factor	No hits found	ER digestion	Monomorphic	-	-	-
**Vf_Mt1g076600**[b]	MTR_1g076600	Cyclin-D-binding Myb-like transcription factor	Locus_12353_Transcript_2/3_Confidence_0.600_Length_1242	ER digestion	Monomorphic	-	-	-
**Vf_Mt1g076780**[b]	MTR_1g076780	WD40 repeat protein	Contig15218	ER digestion	Unreliable data for genotyping	-	-	-
**Vf_Mt1g077640**[b]	MTR_1g077640	bHLH transcription factor	No hits found	ER digestion	Polymorphic	-	-	X (BamHI)
GLPSNP[c]	MTR_1g079490	Germin-like protein subfamily 1 member 16	-	KASPAR assay	Polymorphic	X	X	X
Vf_Mt1g079520[c]	MTR_1g079520	LRR receptor-like kinase	-	KASPAR assay	Monomorphic	-	-	-
Vf_Mt1g079870[c]	MTR_1g079870	Hypothetical protein	-	KASPAR assay	Polymorphic	X	-	-
Vf_Mt1g079930[c]	MTR_1g079930	Activating signal cointegrator	-	KASPAR assay	Monomorphic	-	-	-
Vf_Mt1g080150[c]	MTR_1g080150	RNA-binding protein 42	-	KASPAR assay	Monomorphic	-	-	-
Vf_Mt1g081290[c]	MTR_1g081290	Zeta-carotene desaturase	-	KASPAR assay	Monomorphic	-	-	-
Vf_Mt1g082210[c]	MTR_1g082210	Gibberellin receptor GID1c-like protein	-	KASPAR assay	Polymorphic	-	X	X
Vf_Mt1g082420[c]	MTR_1g082420	Thylakoid lumen 18.3 kDa protein	-	KASPAR assay	Polymorphic	X	-	-
**Vf_Mt1g082480**[b]	MTR_1g082480	bHLH transcription factor	No hits found	ER digestion	Unreliable data for genotyping	-	-	-
Vf_Mt1g082580[c]	MTR_1g082580	Serine/Threonine kinase family protein	-	KASPAR assay	Polymorphic	-	-	X
Vf_Mt1g083230[c]	MTR_1g083230	WD repeat phosphoinositide-interacting-like protein	-	KASPAR assay	Monomorphic	-	-	-
Vf_Mt1g083450[c]	MTR_1g083450	Hypothetical protein	-	KASPAR assay	Monomorphic	-	-	-
Vf_Mt1g083460[c]	MTR_1g083460	60S ribosomal protein L18-3	-	KASPAR assay	Polymorphic	X	X	-
Vf_Mt1g083960[c]	MTR_1g083960	Calreticulin	-	KASPAR assay	Monomorphic	-	-	-
**Vf_Mt1g084160**[b]	MTR_1g084160	bHLH transcription factor	Contig23306	ER digestion	Unreliable data for genotyping	-	-	-
Vf_Mt1g085040[c]	MTR_1g085040	MYB transcription factor	-	KASPAR assay	Polymorphic	X	X	-
**Vf_Mt1g086180**[b]	MTR_1g086180	MYB related transcription factor	Contig1005	ER digestion	Unreliable data for genotyping	-	-	-
GLIP133SNP[c]	MTR_1g086340	Polyketide cyclase/dehydrase and lipid transporter	-	KASPAR assay	Monomorphic	-	-	-
**Vf_Mt1g086790**[b]	MTR_1g086790	WRKY family transcription factor	Locus_9490_Transcript_1/1_Confidence_1.000_Length_898	ER digestion	ER not available	-	-	-
Vf_Mt1g086810[c]	MTR_1g086810	Heme-binding protein	-	KASPAR assay	Monomorphic	-	-	-
**Vf_Mt1g087120**[b]	MTR_1g087120	MYB related transcription factor	Locus_40110_Transcript_1/1_Confidence_1.000_Length_274	ER digestion	Unreliable data for genotyping	-	-	-
LG031SNP[c]	MTR_1g087760	Kynurenine-oxoglutarate transaminase-like protein	-	KASPAR assay	Monomorphic	-	-	-
LG038SNP[c]	MTR_1g087900	Fumarate hydratase	-	KASPAR assay	Polymorphic	X	-	-
Vf_Mt1g088190[c]	MTR_1g088190	Endoglucanase 17	-	KASPAR assay	Polymorphic	X	X	-
Vf_Mt1g089980[c]	MTR_1g089980	No hit found	-	KASPAR assay	Polymorphic	X	-	-
Vf_Mt1g114560[c]	MTR_1g114560	Riboflavin kinase	-	KASPAR assay	Monomorphic	-	-	-
Vf_Mt1g116110[c]	MTR_1g116110	Protoheme IX farnesyltransferase	-	KASPAR assay	Polymorphic	-	X	-
Vf_Mt1g116230[c]	MTR_1g116230	Leukotriene A-4 hydrolase-like protein	-	KASPAR assay	Monomorphic	-	-	-
Vf_Mt1g116810[c]	MTR_1g116810	Prolyl 4-hydroxylase alpha-like protein	-	KASPAR assay	Monomorphic	-	-	-
GLIP065[a]	MTR_4g021350	Medicago truncatula clone mth2-14c14	-	ER digestion	Polymorphic	-	-	X (AciI)
Vf_Mt4g080370[c]	MTR_4g080370	No results NCBI	-	KASPAR assay	Polymorphic	X	X	-
Vf_Mt5g042440[c]	MTR_5g042440	LysM-domain receptor-like kinase	-	KASPAR assay	Polymorphic	X	-	-
GLIP651[a]	MTR_5g085850	NRT1/ PTR FAMILY	-	ER digestion	Monomorphic	-	-	-
Vf_Mt7g035110[c]	MTR_7g035110	RXT3-like protein	-	KASPAR assay	Polymorphic	X	-	-
Vf_Mt7g100500[c]	MTR_7g100500	Serine/threonine protein kinase PBS1	-	KASPAR assay	Polymorphic	X	X	X
REPSNP[c]	MTR_8g068875	Medicago truncatula protein RAE1	-	KASPAR assay	Monomorphic	-	-	-

*Restriction Enzymes used for genotyping are between brackets

[a] EST (Ellwood et al. 2008; Satovic et al. 2013)

[b] New gene-based markers developed in this study (in bold)

[c] KASPar markers (Webb et al. 2016)

The bHLH transcription factor VfTT8 underlies *zt2, the locus determining zero tannin content in faba bean (Vicia faba L.)*

Natalia Gutierrez[1*], CM Avila[1], Ana M. Torres[1]
[1] Área de Genómica y Biotecnología, IFAPA-Centro Alameda del Obispo, Apdo 3092, E-14080 Córdoba, Spain.
*Corresponding author e-mail: natalia.gutierrez.leiva@juntadeandalucia.es

Supplementary Table S2. Characteristics of the 23 *Medicago truncatula* markers associated with anthocyanin expression and designed in this study. The information about marker name and type, gene description, orthologous sequence in faba bean, primer sequence, annealing temperature , locus name and product size of the amplicon is shown.

Marker name	Marker type	Medicago locus	Gene description	BLASTn_VF_Transcriptome (Ocaña et al. 2016)	Primer Sequence (5´-3´)	Ta	PCR product size (bp)
Vf_Mt1g043080	SNP	MTR_1g043080	MYB relation transcription (MYB)	Contig15498	TAGGGAACAGGTGGTCAGCA<br>TGCACCCCTTCACTTGTTGTA	55°C	667
Vf_Mt1g045610	ALP	MTR_1g045610	R2R3-MYB Transcription factor	No hits found	TGGACTGCTGAAGAGGATAGGTT<br>ATTATGCTTTCTTCTTGTGGTGTTA	57°C	719 (Vf6)/ 574 (zt2)
Vf_Mt1g070180	SNP	MTR_1g070180	bHLH transcription factor	Locus_34012_Transcript_1/1_Confidence_1.000_Length_809	CCAAAGCATAACTAAGCATGCTGAT<br>TCCATTGCCCAACTTGAACCC	60°C	201
Vf_Mt1g070455	SNP	MTR_1g070455	WRKY family transcription factor	Locus_48067_Transcript_1/1_Confidence_1.000_Length_887	CGTGGAGGAAGTACGGGCA<br>CAAGCACCTCTCGACATGCTTC	58°C	550
Vf_Mt1g070870	SNP	MTR_1g070870	bHLH transcription factor	Contig4759	GAGCTGCCATGTTGGATGAAATTG<br>GGATATCAGTTACCAGTGGGGC	60°C	336
Vf_Mt1g072130	SNP	MTR_1g072130	PHDfingerprotein,putative	Locus_31961	TTCTAGATCCAATGGTCGTAGTGT<br>CCTTGTGTCATCGTCTCATTGTG	54°C	639
Vf_Mt1g072140	SNP	MTR_1g072140	transducin/WD40 repeatprotein	No hits found	TGCGTCAAATTCTCCAACGA<br>AGAGTAGCAACAAGTATAAATTTCCC	54°C	554
Vf_Mt1g072320	SNP	MTR_1g072320	Transcription factor TT8	No hits found	GTTGCCAGGAAAGGCATATGC<br>CTTATCAGTTGTGCCAATCTCAACG	57°C	690 (1)
					AAGAAGACACACACTACTCTCAAAC<br>TCAATCTCCAACAAAGCATCACTC	57°C	670 (2)
Vf_Mt1g072530	SNP	MTR_1g072530	Transcription factor bHLH66	Locus_21144	TGCAACATGGCAAACTGATTCA<br>ACTAGTGGTATAGCATTTGAGTCA	60°C	615
Vf_Mt1g073170	SNP	MTR_1g073170	MYB related transcription factor	Locus 29372	CAGGACTGAGAAGAAATGGGAAAAG<br>CATCAAGCAACAAGTCTATGATCCC	58°C	493
Vf_Mt1g073920	SNP	MTR_1g073920	Actin related rpotein ARP4	Contig21805	GAGGGGCAACTTGATGCTGT<br>TCTTCAACTGCAGGAACTAAACCA	56°C	800
Vf_Mt1g074370	SNP	MTR_1g074370	Ethylene-responsive transcription factor	Contig5707	GAATTCAACTATCTCCCCTTCAACG<br>CGGAAGACATCAACAATTGTTCCA	60°C	568
Vf_Mt1g075320	SNP	MTR_1g075320	N-carbamyl-L-amino acid amidohydrolase	Locus_2084_Transcript 2180	CGCTTATTGGCTGGGAGTGA<br>GCTAGGGATACTATTGATTGCTCCA	60°C	820
Vf_Mt1g075740	SNP	MTR_1g075740	COP1-interacting-like protein	Contig16561	GAGGTTGGATTCGGCTGTTTTTC<br>CCACCCTTGTCCATCTGATCTT	56°C	668
Vf_Mt1g076150	SNP	MTR_1g076150	MYB related transcription factor	No hits found	GAGCTCCTTGTTGTGAAAAGATGG<br>CTGCAGCTTTTTCCACACCTTAAC	54°C	320
Vf_Mt1g076600	SNP	MTR_1g076600	cyclin-D-binding Myb-like transcription factor	Locus_12353_Transcript_2/3_Confidence_0.600_Length_1242	GATGACTTCCGCTTGATAGGTGC<br>GCATCGGCGATCCCACC	60°C	352
Vf_Mt1g076780	SNP	MTR_1g076780	WD-40 repeat protein	Contig15218	GGGGAAACTTGCATTTGACATTG<br>CCACATCTGTAGCCAGTATCGAG	64°C	720
Vf_Mt1g077640	SNP	MTR_1g077640	bHLH transcription factor	No hits found	GTGTAGCATCGATTCTTTGTTAGAC<br>AATTATGTCGACTATCTCAAGGAAGAA	56°C	477
Vf_Mt1g082480	SNP	MTR_1g082480	bHLH transcription factor	No hits found	GAAATTGAGAATCAGAGAATGACTCAC<br>GAATACTGTGGAAATGTAAAGAACTCA	56°C	503
Vf_Mt1g084160	SNP	MTR_1g084160	bHLH transcription factor	Contig23306	CTCTCTTCTCTTCCTTTCTCCTCA<br>CGGATTCAGATCTCCACCGC	56°C	584
Vf_Mt1g086180	SNP	MTR_1g086180	MYB related transcription factor	Contig1005	TCTCATGATTCATGTGGCCCTTCT<br>CAAGCATAAGGATTCAGCAAGGTCA	56°C	452
Vf_Mt1g086790	SNP	MTR_1g086790	WRKY family transcription factor	Locus_9490_Transcript_1/1_Confidence_1.000_Length_898	CAGAGAGAACCAAGATTCGCGTT<br>CCCTAAGAAAGGAAGTAGTTGTCCC	56°C	333
Vf_Mt1g087120	SNP	MTR_1g087120	MYB related transcription factor	Locus_40110_Transcript_1/1_Confidence_1.000_Length_274	GGCAAATCTTGAAAGTAGACCGAG<br>GTTGGTTTAAAGGGTGCTTTCATT	58°C	393

(1) Product size obtained with primer pair 1
(2) Product size obtained with primer pair 2

The bHLH transcription factor VfTT8 underlies *zt2, the locus determining zero tannin content in faba bean (Vicia faba L.)*

Natalia Gutierrez[1*], CM Avila[1], Ana M. Torres[1]
[1] Área de Genómica y Biotecnología, IFAPA-Centro Alameda del Obispo, Apdo 3092, E-14080 Córdoba, Spain.
*Corresponding author e-mail: natalia.gutierrez.leiva@juntadeandalucia.es

Supplementary Table S3 Haplotype analysis of the markers flanking the gene zt2 in three faba bean populations. The allele for each phenotype (flower color) and for each gene maker is specified. The grey color indicates the misclassified individuals.

Population	Phenotype F3 family	Vf_Mt1g072320(2)	GLIP245SNP	Vf_Mt1g072140	Vf_Mt7g100500
**MAYA_P1**	**wild type (A)**	**T:T**	**T:T**	**C:C**	**C:C**
**DISCO_P2**	***zt2* (B)**	**C:C**	**C:C**	**A:A**	**T:T**
**1**	**A**	T:T	T:T	C:C	C:C
**2**	**A**	T:C	T:C	C:A	T:C
**3**	**H**	T:C	T:C	C:A	T:C
**4**	**A**	T:T	T:T	C:C	C:C
**5**	**B**	C:C	C:C	A:A	T:T
**6**	**A**	T:C	T:C	C:A	T:C
**7**	**A**	T:C	T:C	C:A	T:C
**8**	**H**	T:C	T:C	C:A	T:T
**9**	**H**	T:C	T:C	C:A	T:T
**10**	**H**	T:C	T:C	C:A	T:C
**11**	**A**	T:T	T:T	C:C	C:C
**12**	**B**	C:C	C:C	A:A	T:T
**13**	**B**	C:C	C:C	A:A	T:T
**14**	**H**	T:C	T:C	C:A	T:C
**15**	**H**	T:C	T:C	C:A	T:C
**16**	**H**	T:C	T:C	C:A	T:C
**17**	**H**	T:C	T:C	C:A	T:C
**18**	**H**	T:C	T:C	C:A	T:C
**19**	**A**	T:T	T:T	C:C	C:C
**20**	**A**	T:T	T:T	C:C	T:C
**21**	**H**	T:C	T:C	A:A	T:C
**22**	**B**	C:C	C:C	A:A	T:T
**23**	**H**	T:C	T:C	C:A	T:C
**24**	**H**	T:C	T:C	C:A	T:C
**25**	**H**	T:C	T:C	C:A	T:C
**26**	**H**	T:C	T:C	C:A	T:T
**27**	**H**	T:C	T:C	C:A	T:C
**28**	**H**	T:C	T:C	C:A	C:C
**29**	**B**	C:C	C:C	C:A	T:T
**30**	**H**	T:C	T:C	C:A	T:T
**31**	**B**	C:C	C:C	A:A	T:T
**32**	**A**	T:T	?	C:A	C:C
**33**	**A**	T:T	T:T	C:A	T:C
**34**	**A**	T:T	T:T	C:A	C:C
**35**	**B**	C:C	C:C	A:A	T:C
**36**	**H**	T:C	T:C	C:A	T:C
**37**	**H**	T:C	T:C	C:A	T:C
**38**	**H**	T:C	T:C	C:A	C:C
**39**	**H**	T:C	T:C	C:A	T:T
**40**	**H**	T:C	T:C	C:A	T:C
**41**	**H**	T:C	T:C	C:A	T:T
**42**	**H**	T:C	T:C	C:A	T:C
**43**	**H**	T:C	C:C	C:A	T:T
**44**	**H**	T:C	T:C	C:A	T:C
**45**	**H**	T:C	T:C	A:A	T:C
**46**	**H**	T:C	T:C	C:A	T:C
**47**	**A**	T:T	T:T	C:C	T:T
**48**	**H**	T:C	T:C	C:A	T:C
**49**	**B**	C:C	C:C	A:A	T:T
**50**	**H**	T:C	T:C	C:A	C:C
Misclassified individuals		3	5	9	17

Population	Phenotype F3 family	Vf_Mt1g072320[1]	Vf_Mt1g072530	GLPSNP	Vf_Mt7g100500
**WIZARD_P1**	**wild type (A)**	**A:A**	**A:A**	**G:G**	**C:C**
**DISCO_P2**	***zt2* (B)**	**G:G**	**C:C**	**C:C**	**T:T**
1	**A**	A:A	A:A	G:G	C:C
**2**	**H**	G:A	C:A	G:C	T:C
**3**	**H**	G:A	C:A	G:C	T:C
**4**	**H**	G:A	C:A	G:C	T:C
**5**	**A**	A:A	A:A	G:G	C:C
**6**	**H**	G:A	C:A	G:C	T:C
**7**	**B**	G:G	C:A	C:C	T:T
**8**	**A**	A:A	C:A	G:G	T:C
**9**	**A**	A:A	A:A	G:G	C:C
**10**	**A**	A:A	A:A	G:C	T:C
**11**	**B**	G:G	C:C	G:C	T:C
**12**	**H**	G:A	C:A	G:C	T:C
**13**	**H**	G:A	C:A	C:C	T:C
**14**	**H**	G:A	C:A	G:C	T:C
**15**	**A**	A:A	A:A	G:G	C:C
**16**	**B**	G:G	C:C	C:C	T:T
**17**	**H**	G:A	C:A	G:C	C:C
**18**	**H**	G:A	C:A	G:C	T:C
**19**	**A**	G:A	C:A	C:C	C:C
**20**	**A**	A:A	A:A	G:C	C:C
**21**	**B**	G:G	C:C	G:C	T:T
**22**	**B**	G:G	C:C	C:C	T:T
**23**	**B**	G:G	C:C	C:C	T:T
**24**	**H**	G:A	C:A	G:C	T:C
**25**	**A**	A:A	A:A	G:G	C:C
**26**	**A**	A:A	A:A	G:G	C:C
**27**	**H**	G:A	C:A	G:C	T:C
**28**	**B**	G:G	C:C	C:C	T:T
**29**	**H**	G:A	C:A	G:C	T:C
**30**	**H**	G:A	C:A	G:C	T:T
**31**	**H**	G:A	C:A	G:C	T:C
**32**	**H**	G:A	C:A	G:C	T:C
**33**	**A**	A:A	A:A	G:C	C:C
**34**	**H**	G:A	C:A	G:C	T:C
**35**	**A**	G:A	C:A	G:C	T:T
**36**	**H**	G:A	C:A	G:C	T:C
**37**	**B**	G:G	C:C	C:C	T:T
**38**	**A**	A:A	A:A	G:G	C:C
**39**	**H**	G:A	C:A	G:C	T:C
**40**	**A**	A:A	A:A	G:C	T:C
**41**	**H**	G:A	C:A	G:C	T:C
**42**	**H**	G:A	C:A	G:C	T:C
**43**	**B**	G:G	C:C	C:C	T:T
**44**	**B**	G:G	C:C	C:C	T:T
**45**	**H**	G:A	C:A	G:C	T:C
**46**	**A**	A:A	A:A	G:G	T:C
**47**	**H**	G:A	?	G:C	T:T
**48**	**A**	A:A	A:A	G:G	C:C
**49**	**A**	A:A	A:A	G:G	C:C
**50**	**A**	A:A	A:A	G:G	T:T
**51**	**B**	G:G	C:C	C:C	T:T
**52**	**B**	G:G	C:C	C:C	T:T
**53**	**H**	G:A	C:A	G:C	T:C
**54**	**B**	G:G	C:C	C:C	T:T
**55**	**H**	G:A	C:A	G:C	T:C
**56**	**B**	G:G	C:A	C:C	T:T
**Misclassified individuals**		**2**	**6**	**9**	**10**

	Phenotype RIL individual	Vf_Mt1g072320[(1)]	Vf_Mt1g072530	Vf_Mt1g077640	Vf_Mt7g100500
**VF6_P1**	**wild type (A)**	**A:A**	**C:C**	**A:A**	**C:C**
**ZT2_P2**	***zt2* (B)**	**G:G**	**G:G**	**G:G**	**T:T**
**1**	**A**	A:A	C:C	A:A	C:C
**2**	**B**	G:G	G.G	G:G	T:T
**3**	**A**	A:A	C:C	A:A	C:C
**4**	**A**	A:A	C:C	A:A	T:T
**5**	**B**	G:G	G.G	G:G	T:T
**6**	**A**	A:A	C:C	A:A	C:C
**7**	**B**	G:G	G.G	A:A	T:T
**8**	**B**	G:G	G.G	G:G	T:T
**9**	**A**	A:A	C:C	A:A	C:C
**10**	**A**	A:A	C:C	G:G	C:C
**11**	**A**	A:A	C:C	A:A	C:C
**12**	**A**	A:A	C:C	A:A	C:C
**13**	**B**	G:G	G.G	G:G	C:C
**14**	**A**	A:A	G:C	G:G	T:T
**15**	**A**	A:A	C:C	A:A	T:T
**16**	**A**	A:A	C:C	A:A	C:C
**17**	**B**	G:G	G.G	G:G	T:T
**18**	**A**	A:A	C:C	A:A	C:C
**19**	**A**	A:A	C:C	A:A	C:C
**20**	**B**	G:G	G.G	A:A	T:T
**21**	**A**	A:A	C:C	G:G	C:C
**22**	**B**	G:G	G.G	G:G	C:C
**23**	**B**	G:G	?	G:G	?
**24**	**A**	A:A	C:C	A:A	T:T
**25**	**A**	G:A	G:C	A:A	C:C
**26**	**A**	G:A	C:C	G:G	C:C
**27**	**B**	G:G	G.G	G:G	T:T
**28**	**B**	G:G	G.G	A:A	T:T
**29**	**A**	A:A	C:C	A:A	C:C
**30**	**A**	A:A	C:C	A:A	C:C
**31**	**A**	A:A	C:C	A:A	T:C
**32**	**A**	A:A	C:C	A:A	T:T
**33**	**B**	G:G	G.G	G:G	T:T
**34**	**A**	A:A	C:C	A:A	C:C
**35**	**A**	A:A	C:C	A:A	C:C
**36**	**A**	A:A	C:C	A:A	C:C
**37**	**A**	A:A	C:C	A:A	C:C
**38**	**A**	A:A	C:C	A:A	T:C
**39**	**A**	A:A	G:C	A:A	T:C
**40**	**B**	G:G	G.G	G:G	T:C
**41**	**B**	G:G	G.G	G:G	C:C
**42**	**B**	G:G	G.G	G:G	T:T
**43**	**A**	A:A	C:C	A:A	C:C
**44**	**A**	A:A	G:C	A:A	T:C
**45**	**A**	A:A	C:C	A:A	C:C
**46**	**A**	A:A	C:C	A:A	C:C
**47**	**A**	A:A	C:C	A:A	C:C
**48**	**B**	G:G	G.G	G:G	C:C
**49**	**B**	G:G	G.G	G:G	C:C
**50**	**B**	G:G	G.G	A:A	C:C
**51**	**A**	A:A	C:C	A:A	C:C
**52**	**B**	G:G	G.G	G:G	T:T
**53**	**A**	A:A	C:C	G:G	T:C
**54**	**B**	G:G	G.G	G:G	T:T
**55**	**A**	A:A	C:C	A:A	C:C
**56**	**B**	G:G	G.G	G:G	T:T
**57**	**B**	G:G	G.G	G:G	T:T
**58**	**B**	G:G	G.G	G:G	T:C
**59**	**B**	G:G	G.G	G:G	T:T
**60**	**B**	G:G	G.G	G:G	T:T
**61**	**A**	A:A	C:C	A:A	T:C
**62**	**B**	G:G	G.G	G:G	T:T
**Misclassified individuals**		**2**	**5**	**8**	**20**
